# Deciphering the Intricate Interplay in the Framework of Antibiotic-Drug Interactions: A Narrative Review

**DOI:** 10.3390/antibiotics13100938

**Published:** 2024-10-05

**Authors:** Andrei-Flavius Radu, Simona Gabriela Bungau, Raluca Anca Corb Aron, Alexandra Georgiana Tarce, Ruxandra Bodog, Teodora Maria Bodog, Ada Radu

**Affiliations:** 1 Doctoral School of Biological and Biomedical Sciences, University of Oradea, 410087 Oradea, Romania; andreiflavius.radu@uoradea.ro (A.-F.R.); bodogruxandra@gmail.com (R.B.); bodogteodora@gmail.com (T.M.B.); 2 Department of Preclinical Disciplines, Faculty of Medicine and Pharmacy, University of Oradea, 410073 Oradea, Romania; raluca14@yahoo.com; 3 Department of Pharmacy, Faculty of Medicine and Pharmacy, University of Oradea, 410028 Oradea, Romania; 4 Medicine Program of Study, Faculty of Medicine and Pharmacy, University of Oradea, 410073 Oradea, Romania; tarce.alexandrageorgiana@student.uoradea.ro

**Keywords:** antibiotic-drug interactions, antibiotics, drug-drug interactions, antibiotic-antibiotic interactions, drug interaction checker, geriatric population, pharmacokinetics, pharmacodynamics

## Abstract

Drug interactions are a significant and integral part of the concept of medication-related adverse events, whether referring to potential interactions or those currently observed in real-world conditions. The high global consumption of antibiotics and their pharmacokinetic and pharmacodynamic mechanisms make antibiotic-drug interactions a key element that requires continuous study due to their clinical relevance. In the present work, the current state of knowledge on antibiotic-drug interactions, which are less studied than other drug-drug interactions despite their frequent use in acute settings, has been consolidated and updated. The focus was on the interactions of the commonly used antibiotics in clinical practice, on the characteristics of the geriatric population susceptible to interactions, and on the impact of online drug interaction checkers. Additionally, strategies for optimizing the management of these interactions, including spacing out administrations, monitoring, or avoiding certain combinations, are suggested. Sustained research and careful monitoring are critical for improving antibiotic safety and efficacy, especially in susceptible populations, to enhance precision in managing antibiotic-drug interactions.

## 1. Introduction

Antibiotics, strictly referring to the semisynthetic, synthetic, or natural compounds used in the therapeutic management of bacterial infections, are essential pharmacological agents with multiple uses in various domains targeting the prevention and treatment of symptomatic manifestations or complications resulting from infections of a bacterial nature [1,2].

Evidence from the scientific literature emphasizes the significant role of antibiotics in reducing the morbidity and mortality associated with infectious diseases over time [3]. Statistical data on global antibiotic consumption, which has significantly increased over time in line with the continuous emergence and variability of infectious diseases, also supports the huge impact of antibiotics as therapeutic interventions [4,5].

Recent clinical trial data have facilitated the European Medicines Agency’s approval for the 2024 market release of Emblaveo^®^ (a novel aztreonam-avibactam combination), specifically indicated for managing complicated intra-abdominal and urinary tract infections, nosocomial pneumonia, and infections by aerobic Gram-negative bacteria with limited treatment alternatives [6]. Additionally, the United States Food and Drug Administration has approved Zevtera^®^ (ceftobiprole medocaril sodium for injection) for use in adults with bloodstream infections caused by *Staphylococcus aureus*, including cases of infective right-sided endocarditis [7].

With the increase in global utilization and the major beneficial impact on the management of bacterial infections counterbalanced by several unmet needs that have gradually increased, the emerging concept of the ideal antibiotic has come to the attention of the scientific community. In addition to possessing bactericidal or bacteriostatic properties, an optimal antibiotic should exhibit several key characteristics: high selectivity to target the pathogenic bacteria with minimal impact on host cells, physicochemical stability to ensure appropriate storage, biological stability to assure the drug’s efficacy during delivery, adequate water solubility for efficient transport through body fluids to the site of infection, affordability, a slow resistance development, a broad-spectrum bactericidal activity, no teratogenic effects, and no drug-drug or food-drug interactions [8,9].

Nevertheless, the notion of an ideal antibiotic remains entirely theoretical, as preclinical studies and clinical practice reveal that no antibiotic meets all the ideal criteria, and instances of antibiotic failure are frequently observed. Among the various causes, antibiotic resistance is the most extensively studied by the scientific community [10,11]. The emergence of antibiotic resistance has abruptly evolved on a global scale, exceeding earlier expectations by spreading between countries at a faster rate. Superbugs and multidrug-resistant bacteria are currently widespread in numerous places across the globe. Furthermore, there is strong evidence to suggest that the extensive and incorrect administration of antibiotics during the last 80 years has played a major role in the substantial rise of antibiotic resistance [11].

Although antibiotic resistance poses a significant global health challenge, imposing a substantial burden on healthcare systems and projected to cause 10 million deaths annually by 2050 if the current trend of misuse persists, it is not the sole factor contributing to antibiotic failure [12]. Factors contributing to antibiotic failure include also pathogen colonization via biofilm formation, immune system dysfunctions, genetic defects [13], the development of gut dysbiosis due to antibiotic therapy [14], delays in initiating treatment, extended intervals between doses, increased hepatic or renal clearance, inappropriate dosage adjustments relative to body weight [15], and interactions between antibiotics and food [16] or other drugs [17]. Most of the antibiotic-drug interactions observed so far are primarily due to their enzymatic inhibitory or inducing effects on CYP450 enzymes (pharmacokinetic alterations) [18], as well as the additive effects that prolong the QT interval, increasing the risk of torsades de pointes (pharmacodynamic alteration) [19].

Approximately 2.8% of hospital admissions are attributed directly to drug-drug interactions (DDIs) [20]. Astemizole, cisapride, mibefradil, and terfenadine were withdrawn from the market or had their use restricted due to the failure to translate available research into appropriate prescribing adjustments, resulting in dangerous drug interactions when co-prescribed with other interacting medications [21].

Antibiotics are among the most prescribed medications due to the prevalence of infectious diseases. Despite their widespread use, there is a limited body of scientific literature addressing antibiotic-drug interactions, primarily because concomitant medications are often overlooked, given the typically short duration of antibiotic treatments [22]. However, contexts have been identified in which interactions between the antibiotic (e.g., clarithromycin) and the victim drug (e.g., colchicine) occurred after a single dose [23]. This research gap is significant because, while most drug interactions are not severe, some can be life-threatening, making their evaluation and the centralization of updated scientific knowledge essential.

The management of DDIs varies across different patient populations, from pediatrics to geriatrics, and contributes to the substantial costs of medication-related adverse events (MRAEs), which exceed EUR 79 billion in the European Union (EU) and EUR 89 billion in the United States [24]. Therefore, this underscores the significant economic impact and highlights the necessity for effective management of all MRAEs, including DDIs.

A thorough understanding of both pharmacokinetic and pharmacodynamic mechanisms underlying drug interactions is essential for effective healthcare. Additionally, particular attention should be given to special populations, especially elderly patients, who are increasingly numerous and exhibit a higher susceptibility to infections compared to the general adult population, and the prevalence of DDIs in this category varies from 42.5% to 54.4% [25].

As new drugs emerge, and metabolic enzymes and transporters are identified, the complexity of drug interactions in infectious diseases increases [22]. The increasing intricacy, combined with adaptive resistance in pathogens and gaps in research due to limited data, underscores the need for ongoing research in this domain.

The significant risks, which can be life-threatening in certain medical contexts, associated with antibiotic-drug interactions and even antibiotic-antibiotic interactions, coupled with their complex mechanisms and the potential increase in number as new molecules and drug combinations are discovered, have led to a continuous and substantial accumulation of information. To accelerate the process for clinicians to identify potentially harmful drug interactions and improve healthcare systems, drug interaction checker databases have been developed. While these databases are highly useful, particularly for identifying contraindications and major interactions, they provide only indicative value and must be confirmed by a specialist [26].

The present narrative review aims to update the current understanding of antibiotic-drug interactions through a unique approach that consolidates the latest data on the clinical implications of these interactions, their proper management, and the specific considerations for geriatric populations. Furthermore, a detailed and distinct examination of the implications of online interaction checker tools, specifically targeting antibiotic-drug and antibiotic-antibiotic interactions, has been conducted to enhance the current state of the art. This review addresses the research gap created by the limited number of existing publications, which is due to the generally short duration of antibiotic use, by providing a comprehensive yet distinctive contribution to the scientific literature on this topic.

## 2. Methodology of Research

The present paper selects, filters, evaluates, and centralizes scientific publications targeting antibiotic topicality, with a strong focus on their involvement in drug interactions. In this respect, a comprehensive search was performed using informative source databases with a large coverage in terms of medical topicality, targeting antibiotics, and valuable based on their bibliometric parameter values (i.e., PubMed, SpringerLink, ScienceDirect, and Web of Science).

**Figure 1:**
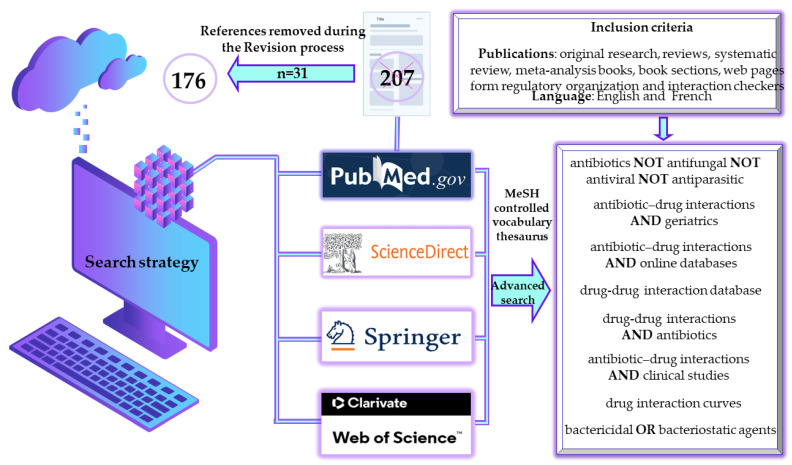
Methodological strategies for selecting literature on antibiotics-drug interactions. MeSH, medical subject heading.

**Figure 2:**
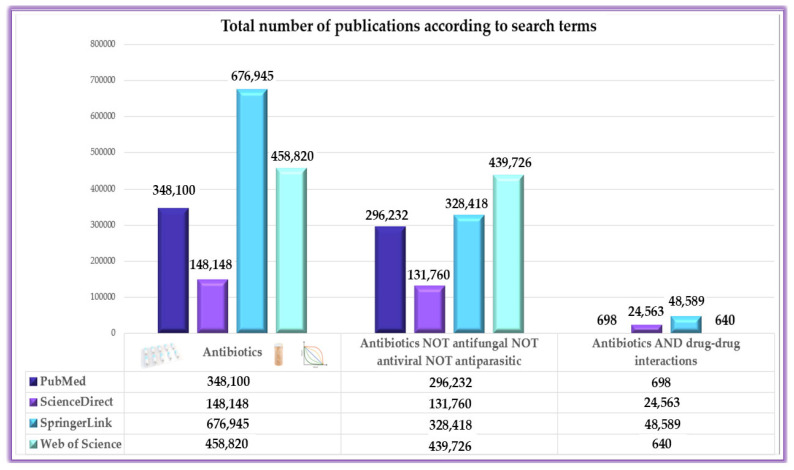
Variations in publication volume across search terms, highlighting the limited focus on antibiotic interactions.

The working methodology included a predefined algorithm to generate results exclusively in the field under present evaluation (Figure 1). Furthermore, the methodological analysis also includes an evaluation based on Boolean operators (i.e., NOT, OR, AND) to emphasize that although studies with antibiotics in the foreground are numerous, being approached from many perspectives, publications evaluating their DDIs are significantly fewer (Figure 2), leading to an area of insufficient research that requires more extensive approaches. However, it can be seen from the results generated that the term ‘antibiotic’ in some cases includes in a broad sense also actions on fungi, viruses and parasites.

Publications written in languages other than English and French, those which were not highly informative or relevant to the current paper’s objective, and those that did not fall under the categories of scientific articles, books, or web pages from international health regulatory and advisory organizations’ databases, were excluded during the scientific literature screening phase. A total of 176 references, predominantly from the last 5 years, were selected, evaluated, and cited to validate the information presented in this narrative review.

## 3. Overview of Antibiotics

The advent of antibiotics marked a seminal advancement in 20th-century medicine [27]. While antibiotics were initially groundbreaking in their efficacy, ongoing enhancement of antibiotic therapy is crucial due to the rapid adaptive resistance mechanisms of bacteria [28,29], even with the diverse array of available antibiotic classes [30]. Consequently, extensive research in this field is essential to address the growing challenge of bacterial resistance and antibiotic failure. A frequent re-evaluation and updating of antibiotic therapy management is therefore necessary, given the numerous classes of antibiotics, some with numerous representatives, the different mechanisms of action that must be adapted to the desired application, the different routes of administration that are related to the chemical structure of antibiotics, etc. [22,31,32]. Moreover, antibiotics belonging to the same structural class typically exhibit comparable effectiveness, toxicity profiles, and probability of causing allergic responses [33,34].

Antibiotics can be categorized in various ways based on diverse parameters essential for the specific type of analysis conducted [33]. The most widely used classifications of antibiotics are based on their origin, mechanisms of action, and response. Natural antibiotics are substances that are synthesized through the secondary metabolic pathways of microorganisms [35]. The compounds in question are synthesized exclusively when required and are not needed for microbial viability. Notable instances of naturally occurring antibiotics include streptomycin [36] and penicillin [37].

The unmet needs related to the utilization of natural antibiotics have led to the development and subsequent authorization of multiple synthetic antibiotics for the management of infectious illnesses. Synthetic antibiotics generally exhibit greater toxicity against bacteria while producing beneficial effects more quickly than natural antibiotics [38]. Furthermore, antibiotics can be classified according to their ability to kill bacteria (i.e., bactericidal) or inhibit their growth (i.e., bacteriostatic). The concentration of the administered antibiotic mostly determines this distinction, suggesting that the antibiotics can exert both effects [39].

Antibiotics exert their effects through various mechanisms. Macrolides (e.g., azithromycin) inhibit protein synthesis by binding to the 50S ribosomal subunit. β-Lactams like aztreonam, imipenem, and penicillin G disrupt bacterial cell wall synthesis. Cephalosporins (e.g., ceftazidime) also target cell wall synthesis. Aminoglycosides (e.g., gentamicin) inhibit protein synthesis by acting on the 30S ribosomal subunit, while glycopeptides like vancomycin block peptidoglycan integration in the cell wall. Polypeptides, including colistin and bacitracin, damage bacterial membranes or inhibit cell wall biosynthesis. Fluoroquinolones (e.g., ciprofloxacin) inhibit DNA gyrase, and oxazolidinones like linezolid block protein synthesis via the 50S subunit. Nitroimidazoles and nitrofurans, such as metronidazole and nitrofurantoin, disrupt DNA replication. Lincosamides (e.g., clindamycin) inhibit protein synthesis, while amphenicols (e.g., chloramphenicol) bind to the 50S ribosomal subunit. Pleuromutilins like lefamulin also inhibit the 50S subunit. Rifamycins (e.g., rifampicin) inhibit RNA synthesis, and sulfonamides (e.g., sulfamethoxazole) block folic acid synthesis. Polyketides like tetracycline inhibit the 30S subunit, while D-cycloserine, fosfomycin, and fidaxomicin inhibit enzymes involved in cell wall and RNA synthesis [30,40,41].

At optimal concentrations, penicillins, cephalosporins, vancomycin, carbapenems, monobactams, fluoroquinolones and aminoglycosides are considered bactericidal antibiotics. By contrast, antibiotics such as chloramphenicol, macrolides, linezolid, trimethoprim, and sulphonamides exhibit a bacteriostatic effect [32,42].

Currently, there are more than 400 antibacterial medications on the pharmaceutical market, encompassing natural, semisynthetic, and completely synthetic antibiotics, with most of them being widely accessible. Penicillins, cephalosporins, quinolones, macrolides, and tetracyclines are the antibiotics most commonly prescribed and distributed [32].

In addition to those frequently used, recent advancements in antibiotic development over the past decade, while still insufficient, have yielded notable outcomes, including the approval and commercialization of the following drugs: pazufloxacin mesylate, finafloxacin, tedizolid, delamanid, ceftozolan-tazobactam (2014), zabofloxacin hydrochloride, cefatazidime-avibactam (2015), nemonoxacin (2016), delafloxacin megulmine, ozenoxacin, meropenem-varbobactam (2017), lascufloxacin hydrochloride, lefamulin, nadifloxacin, cefiderocol, alalevonadifloxacin mesylate, sarecycline hydrochloride, imipenem-cilastatin-relebactam, pretomanid (2019) [43], contezolid (2021) [44], sulbactam-durlobactam, taurolidine (2023) [45], aztreonam-avibactam [6], ceftobiprole medocaril sodium [46,47], cefepime-enmetazobactam (2024) [47].

The frequent occurrence of antibiotic failure, the excessive use of antibiotics leading to heightened bacterial resistance, and the annual approval of new anti-infective agents necessitate the ongoing updating, expansion, and development of data regarding potential antibiotics-drug interactions with negative clinical consequences.

## 4. Antibiotic-Drug Interactions

A deeper understanding of the correlations between key data concerning antibiotics (i.e., mechanism of action, pharmacokinetic and pharmacodynamic parameters, possibilities and patterns of interaction with other drugs in case of combined administrations) and bacteria (i.e., resistance mechanisms, replication cycles), together with their clinical implications, is essential for optimizing therapeutic strategies.

Critically ill patients are often on multiple medications and are at high risk for drug interactions. Antibiotics are widely used in intensive care units because these patients are susceptible to infections and have impaired immune systems. The serious conditions requiring multiple medications in the intensive care unit increase the likelihood of drug interactions. Therefore, understanding antibiotic-drug interactions is essential, as they can impact antibiotic efficacy and the occurrence of adverse effects [48,49].

MRAEs have been approximately ranked as the fourth to sixth most prevalent cause of mortality globally [50]. MRAEs are a global public health issue that requires monitoring because of their significant influence on quality of life, morbidity, life expectancy, mortality, and expenses for healthcare. DDIs significantly contribute to MRAEs, along with improper dosing or treatment time span, off-label use, or utilization in contraindicated contexts [51,52].

The financial expense of treating preventable MRAEs is excessive, reaching billions of dollars each year [53,54]. Furthermore, projections indicate that the expense associated with each preventable MRAE will surpass that of non-preventable MRAEs [52].

Evaluations show that 60% of MRAEs are preventable. DDIs are a significant contributor to avoidable MRAEs [55]. The rising prevalence of patients with multiple chronic conditions and the complex nature of medications have resulted in a widespread use of multiple medications, known as polypharmacy. This context can lead to a higher risk of potential DDIs [51]. Current DDIs are determined through clinical evidence, including symptoms or laboratory testing findings. As a result, the occurrence of actual DDIs is significantly less frequent compared to potential DDIs [56].

Identifying potential DDIs has always been a challenging task in clinical research and drug design [57]. Medical data found in the scientific literature highlight the importance of addressing this issue, as there are many unmet needs in terms of understanding and managing potential interactions. Alarmingly, the results of an evaluation showed that a significant 52% of the 255 pharmacies evaluated in Illinois were unable to prevent the distribution of medications that were known to have risky interactions with other drugs. Out of five prescriptions, three of them contained antimicrobials. The combinations of these antimicrobials included, among others, ciprofloxacin-tizanidine, clarithromycin-statin, and clarithromycin-ergotamine. It is essential to point out that these combinations have the potential to cause significant harm [58].

A retrospective study was conducted to analyze specific medical data from a university hospital in the period 2011–2020. The study aimed to examine the distribution of MRAEs and evaluate potential DDIs among MRAEs involving multiple suspected drugs. The study identified the incidence of MRAEs resulting from actual DDIs and described the MRAEs caused by these interactions. The report indicated a total of 1803 MRAEs, out of which 156 were MRAEs associated with potential DDIs. Specifically, there were 100 MRAEs with one potential DDI and 56 MRAEs with multiple potential DDIs. Upon conducting verification and validation, a grand total of 105 actual DDIs were formally validated. In addition, the study found that systemic antimicrobial medicines were the subgroup most commonly associated with MRAEs. Specifically, 14.53% of these MRAEs were classified as severe, and 39.32% were considered preventable. Levofloxacin was the second most frequently occurring medicine responsible for MRAEs, with moxifloxacin, azithromycin, and cefuroxime also being among the top 10 drugs associated with MRAEs. When evaluating the severity of MRAEs, levofloxacin ranked third as a causative drug, and the combination of cefoperazone-sulbactam was also among the top 10 [51].

Anti-infective medications, accounting for 45.87% of the total 1780 drugs administered, were the primary drug classes responsible for causing clinically significant potential DDIs, according to a cross-sectional analysis. The most commonly identified interactions include ciprofloxacin, rifampicin, clarithromycin, erythromycin, co-trimoxazole, levofloxacin, meropenem, and ofloxacin as precipitant drugs. Therefore, the research, identification, updating, characterization, and management of antibiotic-drug interactions become essential based on the unmet needs observed in scientific research [59].

Interactions can be characterized in terms of severity by evaluating risk ratings and categorized into classes A (unknown), B (minor), C (moderate), D (major), and X (contraindicated). However, a more important classification of drug interactions pertains to the mechanism and model of interaction, which can be divided into pharmacokinetic and pharmacodynamic [60].

Advancements in pharmacokinetic study findings have greatly enhanced the knowledge of the mechanism behind pharmacokinetic drug interactions in the past few years. As a result, investigators are currently able to estimate the extent of drug interactions for a wide range of drug combinations [61]. The pharmacokinetic effect involves assessing the systemic exposure, which is influenced by modifications in the absorption, distribution, metabolism, and excretion (ADME) of the victim drug, the second item of a drug interaction [62]. Therefore, the implications of drug interactions based on pharmacokinetic mechanisms are multiple and complex, targeting mechanisms, structures, and processes in each ADME stage: absorption (i.e., changes in pH, chelation and adsorption, changes in gastric emptying and intestinal motility, effects of intestinal blood flow, changes in presystemic clearance, cytochrome P450 enzymes, changes in active and passive transport through P-glycoprotein), distribution (i.e., protein binding and displacement), metabolism (i.e., genetic polymorphisms, mechanisms of enzyme induction and inhibition), and elimination (i.e., glomerular filtration, tubular reabsorption, tubular secretion) [58,62].

DDIs may arise not only from pharmacokinetic interactions, but also from pharmacodynamic ones. Pharmacodynamic interactions may occur at the desired site of biological activity, and they appear regardless of drug concentrations in the plasma or total blood. The pharmacodynamic influence involves assessing the degree of synergy, additivity, or antagonism among two drugs. This feature is determined by their impact on either identical or complementary receptor sites. This form of interaction is frequently observed; however, it may not always be acknowledged or classified as such. For instance, a combination of antibiotics and antiviral medicines is sometimes used to enhance their effectiveness or to avoid the development of bacterial resistance. However, pharmacodynamic interactions can also have negative consequences. Instances of such interactions encompass the possibility of seizures when quinolones are taken in conjunction with NSAIDs [58].

Table 1 comprehensively presents evidence-based medical data on relevant and common antibiotic interactions in different settings and therapeutic regimens. As perpetrator drugs, relevant compounds in terms of interaction potential from different classes of antibiotics were selected.

In the context of DDIs, the perpetrator drug is the medication that initiates an interaction by altering the pharmacokinetic or pharmacodynamic properties of another drug. The affected drug, known as the victim drug, undergoes changes in its activity or metabolism as a result of this interaction [83,84].

Quinolones increase the risk of ventricular arrhythmias due to the additive effect of interactions with various pharmacologically active classes, including antiarrhythmics (e.g., amiodarone, propafenone, procainamide), antipsychotics (e.g., atypicals and phenothiazines), antibiotics (e.g., azithromycin, clarithromycin, quinupristin/dalfopristin), antidepressants (e.g., venlafaxine, tricyclic antidepressants), antiemetics (e.g., dolasetron), beta-blockers (e.g., sotalol), antifungals (e.g., fluconazole, posaconazole), antihistamines (e.g., terfenadine), and antimalarials (e.g., chloroquine, quinine, and hydroxychloroquine) [41,64].

Existing evidence indicates that the hazards related to hypoglycemia appear to be more strongly linked to levofloxacin compared to gatifloxacin or ciprofloxacin. A case-control study also demonstrated a marginal elevation in the incidence of hypoglycemia among diabetic individuals who were given levofloxacin. Nevertheless, ciprofloxacin or moxifloxacin did not exhibit the same risks [85].

Aminoglycosides are implicated in a multitude of medication interactions, a majority of which heighten the probability of nephrotoxicity [41]. Numerous observations indicate a greater risk of kidney damage and ototoxicity when aminoglycosides are administered in combination with loop diuretics, as both types of medications are significant causes of hearing loss [41,86,87]. Moreover, these compounds are recognized for their ability to enhance the paralysis caused by neuromuscular blocking drugs. Aminoglycosides have demonstrated the ability to disrupt the release of acetylcholine and produce a curare-like impact on the postsynaptic receptors. These drugs have the ability to stabilize cell membranes and affect the release of acetylcholine by interfering with the movement of calcium ions at the nerve ending [41,88].

The beta-lactam class, comprising penicillins, cephalosporins, carbapenems, and monobactams, consists of numerous drugs. Interactions between beta-lactam antibiotics and other drugs are rather uncommon, although if they are observed, they usually have little impact on clinical outcomes.

However, the following interactions are of major clinical importance: penicillins and cephalosporins with estrogen-based oral contraceptives, warfarin, probenecid, and methotrexate. Moreover, carbapenems should not be administered with cyclosporine, valproic acid, or theophylline [41].

Macrolides typically cause DDIs by inhibiting the CYP3A4 isoenzyme system. Similar to quinolones, macrolides have the potential to lengthen the QT interval in a dose-dependent manner and increase the risk of cardiac rhythm disturbances, particularly when administered in combination with other medicines that also extend the QT interval [89].

Rifampicin belongs to the rifamycin class, which also includes rifabutin and rifapentine. These drugs have a similar mode of action and typically exhibit cross-resistance [90]. Rifampicin is a strong inducer of the 2C8/9 isoenzymes and CYP3A4. The concurrent use of this antibiotic with other medications may modify their metabolism or transportation depending on their role as substrates for P-glycoprotein or cytochrome P450 in the gastrointestinal tract and liver [91]. Due to the stimulation of the metabolism of CYP2C, some medicines that are metabolized by CYP2C9, such as sulfonylurea antidiabetic agents and (S)-warfarin, result in lower levels in the bloodstream. In addition, rifampicin can decrease the levels of non-metabolized medicines (e.g., digoxin) in the bloodstream by activating drug transporters like P-glycoprotein [92].

To prevent colistin-induced nephrotoxicity [93], it is recommended to avoid the combination of colistin with other nephrotoxic medications such as cisplatin, tenofovir, methotrexate, thiazide diuretics, and antiepileptics, as these can cause various forms of kidney damage [94].

Multiple investigations have documented an intensified reduction in blood clotting factors when co-trimoxazole is included in a patient’s treatment concomitant with warfarin. Furthermore, ciprofloxacin, levofloxacin, metronidazole, fluconazole, azithromycin, and clarithromycin are also classified as high-risk for potential interactions with warfarin [95]. Different studies have extensively established the capacity of co-trimoxazole to elevate serum potassium levels. The cause of this hyperkalemia is the ability of trimethoprim to block the apical membrane potential in the distal nephron [96,97].

Vancomycin is an antibiotic with a glycopeptide structure that is still considered one of the most effective treatments for methicillin-resistant *Staphylococcus aureus* infections [98] but also presents some documented interactions. Vancomycin can potentially interact with other nephrotoxic drugs, particularly aminoglycosides. Patients with impaired kidney function have accumulated vancomycin degradation products [99].

Tetracyclines have been observed to interact with a wide range of drugs. Among the most commonly reported interactions, tetracyclines often act as victim drugs, being chelated by magnesium and calcium cations, which reduce their gastrointestinal absorption [100]. However, tetracyclines have also been identified as perpetrator drugs, particularly in interactions with digoxin and methotrexate. Tetracyclines possess the ability to decrease the population of gastrointestinal bacteria that are involved in the biochemical breakdown of digoxin. Consequently, some individuals may experience an increase in digoxin concentrations [41]. Although case reports on the interaction between tetracyclines and methotrexate are limited, it is recommended to avoid tetracycline administration in patients receiving high-dose methotrexate therapy. This precaution is due to the potential of tetracyclines to disrupt the bacterial flora involved in methotrexate metabolism [41,101].

Chloramphenicol has been documented to interact with a range of medications, including anticoagulants, oral hypoglycemic agents, anticonvulsants, other antibiotics, and analgesics-antipyretics. However, the majority of these interactions are based on case reports with a limited patient population [41].

Quinupristin-dalfopristin has been shown in laboratory investigations to strongly hinder the breakdown of drugs by the enzyme CYP3A4. Co-administering quinupristin-dalfopristin with other medications that are mostly metabolized by CYP3A4 may lead to higher levels of the medications in the bloodstream. This might potentially amplify or prolong their desired benefits and potentially increase the occurrence of negative side effects. Additionally, cases of QT interval prolongation have been reported through additive interaction mechanisms [41,58].

Linezolid is an antibiotic from the oxazolidinone class that inhibits monoamine oxidase-A in a mild, competitive manner. Multiple publications have confirmed the occurrence of serotonin syndrome when linezolid is administered alongside selective serotonin reuptake inhibitors such as sertraline, escitalopram, citalopram, and paroxetine [49,102,103,104].

Among the newest antibiotics introduced to the pharmaceutical market, pretomanid and contezolid have, thus far, shown better safety profiles and lower interaction potential than most antibiotics with a history of antibacterial use [105,106]. However, due to their recent introduction, ongoing monitoring and evaluation are essential for future safety assessments.

A comprehensive approach involving physicians [62,107] and pharmacists is necessary to manage, reduce, or avoid antibiotic-drug interactions. Pharmacists, as healthcare professionals, play a crucial role as the final check before the administration of prescribed medications to patients [108,109]. Among the general recommendations for managing potential antibiotic-drug interactions are selecting an alternative antibiotic, spacing the administration by at least two hours, monitoring the serum/plasma concentrations of the victim drugs, and avoiding the combination entirely if possible [110].

Despite a general approach to drug interactions based on the principle of ‘one size fits all’, it is important to acknowledge that physiological variations among specific populations, particularly elderly, may impact the identification and management of antibiotic-drug interactions.

### Antibiotic-Drug Interactions in the Geriatric Population

Elderly vulnerable individuals are highly susceptible to infections, which are strongly linked to increased rates of illness, death, and consecutively healthcare expenses [111]. The management of infections in elderly patients poses major obstacles due to the variability in their immunological function and pharmacodynamic and pharmacokinetic mechanisms [112]. Moreover, the co-existence of multiple chronic conditions and the polypharmacy can significantly elevate the possibility of experiencing negative reactions and DDIs [113,114]. Approximately 16% of elderly patients are predicted to be susceptible to major drug interactions. Consequently, it is essential to comprehend the mechanisms and rationale underlying drug interactions in older individuals, together with their possible outcomes, in order to ensure appropriate management [115].

In line with evidence-based medical studies, the geriatric population experiences alterations in both pharmacodynamic and pharmacokinetic processes. These modifications can affect the fate of antibiotics in the organism, even in a medical context where no drug interaction is suspected. A decrease in stomach acid generation, a reduction in the small intestine’s surface area, and a decrease in gastric motility hinder the process of absorption into the organism in this specific population. Therefore, a variety of pharmacokinetic and physiological factors influence the bioavailability of drugs, including antibiotics such as azithromycin, erythromycin, cefaclor, ceftibuten, sulfonamides, and cefpodoxime proxetil [112,116].

Increases in adipose tissue and plasma alpha-1-acid glycoprotein levels, combined with losses in lean body mass and total body water, alter the distribution pattern. Consequently, the body distributes lipid-soluble drugs such as rifampin, fluoroquinolones, macrolides, oxazolidinones, and tetracyclines more effectively and retains them for a longer duration. Conversely, the body distributes water-soluble drugs less efficiently, leading to increased bloodstream concentrations of aminoglycosides, glycopeptides, and beta-lactams. Furthermore, there is a decrease in the level of free concentration of macrolides, which are bases [112,117]. Various investigations have shown that hypoalbuminemia can exacerbate medication toxicity by increasing the concentration of unbound pharmaceuticals. Certain antibiotics like penicillins, ceftriaxone, sulfonamides, and clindamycin have demonstrated this effect [112].

A decrease in liver blood flow and a decrease in CYP450 enzyme activity primarily influence metabolic processes. This can extend the time taken for the liver to metabolize antibiotics like macrolides and fluoroquinolones [118]. Changes in excretion are characterized by diminished renal perfusion and a decreased glomerular filtration rate, leading to reduced drug elimination, prolonged drug elimination half-life, accumulation of drugs in the bloodstream, elevated levels of drugs in the serum, and an augmented likelihood of drug toxicity for antibiotics such as beta-lactams, daptomycin, glycopeptides, ciprofloxacin, aminoglycosides, levofloxacin, and co-trimoxazole [112,119].

The geriatric population is more susceptible to producing various changes in the action of drugs, particularly those targeting the cardiovascular system and central nervous system [120,121]. The aging process alters the pharmacodynamics of various substances, thereby impacting their pharmacological response. The pharmacological effect of the active substances mostly relies on the number of target receptors and the drug’s affinity for those targets. Pharmacodynamic changes frequently link to receptor-level signaling or signal transduction pathways, or they may link to alterations in the homeostatic process. The aging process has been observed to impact the expression and function of several receptors [120,122].

Although less so than perturbations in pharmacokinetic mechanisms, pharmacodynamic alterations in the geriatric population may also affect the fate of antibiotics in the organism. Pharmacodynamic mechanisms in this setting refer to the various connections between the concentration of antibiotic in the blood and its ability to bind to bacterial antigens. These interactions ultimately result in the inhibition or death of cells, as determined by the minimum inhibitory concentration (MIC) [111,123].

Given the information on pharmacokinetic and pharmacodynamic alterations in this special population, there is a heightened need for increased awareness and an optimized approach to managing DDIs, including those involving antibiotic-drug interactions.

Although the interaction between penicillins and probenecid can be advantageous in certain medical contexts requiring elevated penicillin serum levels, it is crucial to carefully monitor or possibly avoid this combination in elderly patients. In this age group, there is a heightened risk of drug accumulation due to the competitive inhibition of renal elimination [124].

Another challenging drug interaction involves potent CYP metabolism inhibitors, such as macrolides, and midazolam, an optimal substrate for the CYP3A4 isoenzyme. Consequently, the macrolides telithromycin and clarithromycin are anticipated to raise the levels of midazolam in the organism by 200–800%, resulting in the likelihood of experiencing psychomotor side effects. This potential interaction is particularly crucial in geriatric patients and individuals who are susceptible to the impacts of benzodiazepines [125].

The interaction between co-trimoxazole and renin-angiotensin system inhibitors impairs the balance of potassium in the bloodstream, leading to hyperkalemia and potentially causing fatal cardiac events. Elderly patients, those with compromised renal function, and individuals receiving elevated doses of co-trimoxazole are at increased risk for adverse effects. This increased vulnerability is due to age-related physiological changes, impaired drug clearance, and the potential for drug accumulation with higher dosages [126].

Increased levels of procainamide have been determined due to interaction with trimethoprim, potentially leading to heightened toxicity, especially in the elderly population [64].

A multilayered case-control study was conducted to investigate the risk of upper gastrointestinal tract hemorrhage in a geriatric population who were prescribed warfarin alongside antibiotics routinely used for treating urinary tract infections. The results indicate that co-trimoxazole, compared to other commonly prescribed antibiotics, significantly increases the incidence of upper gastrointestinal tract hemorrhage in older patients receiving treatment with warfarin. Physicians should prioritize prescribing alternate antibiotics whenever feasible for patients who have been prescribed warfarin [127].

A statistical investigation examining antibiotic-drug interactions has yielded the following conclusions: concomitant administration of clarithromycin, an antibiotic that inhibits P-glycoprotein, a multidrug efflux pump responsible for digoxin clearance, can lead to an increased risk of digoxin toxicity; in individuals with diabetes who are administered sulfonylureas (e.g., glyburide) the concurrent use of sulfonamide antibiotics poses a risk for hypoglycemia [128].

Although quinolones are involved in numerous drug interactions, studies have not reported conclusive risks associated with their use in the elderly population. The administration of delafloxacin at a dose of 450 mg twice daily was not found to have a notable impact on the pharmacokinetics of midazolam, the parent compound, or its metabolite, the 1-hydroxy form [129]. Research has shown that the geriatric population does not exhibit increased sensitivity to the inhibitory impact of ciprofloxacin on the liver’s breakdown of theophylline. Moreover, studies have shown that older individuals do not exhibit greater sensitivity than younger individuals to the suppressive impact of ciprofloxacin on the liver’s ability to metabolize antipyrine [41].

A cross-sectional study that focused on DDIs in 209 patients over 60 years of age classified the following antibiotic-drug interactions as risk category X and recommended their avoidance: azithromycin-silodosin (risk of increased serum concentration of silodosin) and ciprofloxacin-domperidone (enhanced QT-prolonging effect) [60].

Interactions between macrolides, particularly clarithromycin and erythromycin, and calcium channel blockers, especially those from the nondihydropyridine class (e.g., verapamil), can lead to significant hypotension and shock, according to evaluated data. The incidence of hypotension due to concurrent administration of calcium channel blockers and macrolides seems to be low. However, the probability and severity of adverse effects appear to be higher in older patients and those with more underlying medical conditions [130].

Given the sharp rise in the global population’s age and the presence of multiple health conditions, the chronic use of multiple medications, and a higher likelihood of antibiotic-drug interactions due to their increased vulnerability to infections needing antibiotics, it is crucial to establish effective strategies and optimized management strategies for maintaining the quality of life of elderly patients. However, in addition to this population, other special groups such as neonates, pregnant women, and pediatric patients must also be considered, as data on drug interactions in these populations are considerably more limited.

## 5. Online Tools in Antibiotic-Drug Interactions

Diverse trends in antibiotic usage have been documented across different regions, with some areas experiencing declines while others show increases. Between 2000 and 2015, global consumption of antibiotics rose significantly, with an overall increase of 65%. Notably, this upward trend was especially accentuated in nations classified as low- or middle-income, where the intensity of antibiotic consumption accelerated rapidly [131].

Recent statistical data suggest that penicillins (i.e., amoxicillin, amoxicillin-clavulanic acid), macrolides (i.e., azithromycin), cephalosporins (i.e., cephalexin), and tetracyclines (i.e., doxycycline) are among the most commonly prescribed classes of antibiotics in the United States, whether for respiratory, skin, or urinary bacterial infections, while carefully considering the spectrum of action of each compound [132]. In Europe, in addition to the antibiotics previously mentioned, quinolones are also included. Although a decrease in their prescription and consumption has been observed, they remain among the most widely used classes. In 2017, ciprofloxacin, levofloxacin, norfloxacin, and moxifloxacin constituted 90% of quinolone utilization within the European area [133]. Furthermore, the same antibiotics remain the most frequently used in lower-middle-income countries as well [134,135].

In high-income countries, a slight decline has been observed in antibiotic consumption rates. Since 2019, the average total consumption of systemic antibiotics in the EU has experienced a reduction of 2.5%, suggesting incremental progress towards achieving the EU’s goal of a 20% decrease by 2030. Cephalosporins and other beta-lactams, macrolides, lincosamides, tetracyclines, quinolones, and streptogramins, were the antibiotic classes whose use in the EU community dropped significantly from 2013 to 2022. The above-mentioned antibiotics are also among the most used antibiotics for the treatment of infections. The administration of trimethoprim and sulfonamides resulted in a notable rise in the EU population-weighted average. The average consumption of penicillins in the EU population showed no notable trends. However, the average consumption of quinolones in the hospital sector of the EU population declined substantially between 2013 and 2022, according to Antimicrobial consumption in the EU/EEA (ESAC-Net)—Annual Epidemiological Report for 2022 (at the end of each year, the Report being updated for the previous year) [136]. In this context, rational prescribing, effective strategies to combat bacterial resistance, and optimal antibiotic management, including the prevention and treatment of antibiotic-drug interactions, are essential. Additionally, ongoing digitization in healthcare offers significant benefits for improving the control of antibiotic use.

Addressing DDIs and improving medication management can be significantly enhanced through e-health solutions. These digital tools and services provide valuable support for both healthcare professionals and patients [107].

Assessing interactions between antibiotics and other drugs via drug interaction databases is crucial, particularly given the extensive global utilization of antibiotics. They offer healthcare professionals crucial insights into potential drug interactions, making them indispensable in clinical practice. Accessible information systems can enhance antibiotic management, improving both efficacy and safety by preventing interactions with other medications [137,138,139].

DDI databases or web servers utilize advanced mathematical algorithms and probabilistic models to function. The models employed, which vary in complexity, include the following: Bayesian probabilistic method-based model, collective probabilistic soft logic-based model, deep attention neural network-based drug–drug interaction prediction, deep feed-forward network-based model, gradient boosting-based model, heterogeneous network-assisted inference, integrated action crossing, label propagation-based model, logistic regression-based model, manifold regularized matrix factorization, meta-learning-based model, multi-relational contrastive learning graph neural network, multichannel feature fusion model for multi-typed DDI prediction, network algorithm and matrix perturbation algorithm-based model, positive-unlabeled learning-based model, random forest-based model, and semantic predication-based model [26].

Therefore, based on statistical data provided by international organizations [132,136] and the scientific literature [131,133,140,141], the most commonly prescribed antibiotics worldwide (i.e., amoxicillin, cephalexin, azithromycin, ciprofloxacin, trimethoprim) were selected for screening potential drug interactions using various open access, English-based interaction checker databases (i.e., Drugs.com [142], Medscape [143], WebMD [144], DrugBank [145], and DDInter [146]).

The screening methodology adopted a dual approach, structured around the following algorithm: the initial phase involved identifying interactions for each selected antibiotic individually using the Drugs.com online tool. This tool is distinct in that it allows for the comprehensive generation of all potential interactions associated with a single drug upon entry, whereas the other applications necessitate the input of at least two medications and only assess potential interactions between the specified drugs. The second step involves comparing the major interaction results from Drugs.com with those identified by the other applications under evaluation.

**Figure 3:**
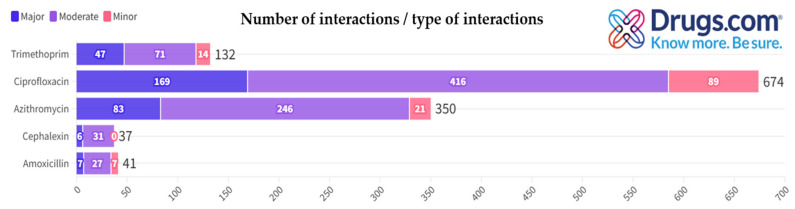
Distribution of antibiotic interactions by severity from Drugs.com.

Figure 3 illustrates the distribution of potential interactions categorized by number and type (i.e., major, moderate, minor) as generated by the Drugs.com application for each antibiotic.

Table 2 includes, in an alphabetical order, only the major potential interactions (due to the extremely high number of interactions identified, especially in the case of moderate and minor ones) for each antibiotic presented in the first column, generated by Drugs.com, when searching for each antibiotic individually. This selection is further justified by the fact that major potential interactions pose the greatest therapeutic risks.

Subsequently, each major interaction identified by Drugs.com was meticulously cross-verified using other online interaction-checker tools. Major interactions observed for ciprofloxacin in the Drugs.com database were cross-verified using the drug interaction checker application from Medscape. Surprisingly, 133 of the 169 major interactions (78.7%) displayed in Drugs.com were characterized differently in Medscape: 58 interactions were classified as moderate, 55 interactions were not identified, and 20 interactions involved compounds not indexed in the Medscape database (Table 3).

Major interactions for azithromycin identified in the Drugs.com database were cross-verified using the drug interaction checker tool from WebMD. Notably, 54 out of 83 major interactions (65%) reported on Drugs.com were classified differently by the WebMD interaction checker: 27 interactions were reclassified as moderate, 4 as minor, 15 interactions were not identified, and 8 interactions involved compounds not indexed in the WebMD database (Table 3).

Major interactions identified for trimethoprim in the Drugs.com database were cross-verified using the drug interaction checker from DrugBank (Table 3). Remarkably, 45 out of the 47 major interactions (95.7%) reported on Drugs.com were classified differently by DrugBank: 21 interactions were categorized as moderate, 14 as minor, 7 were not identified, and 3 involved compounds not listed in the DrugBank database.

**Figure 4:**
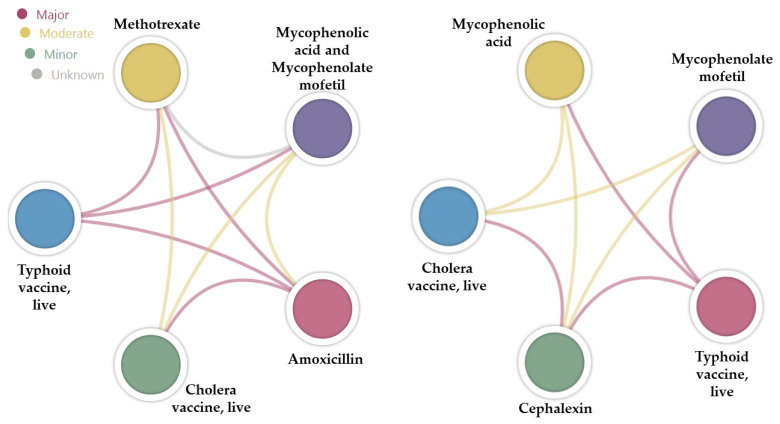
Chord diagrams showing the type of interaction based on the color of the bond between two drugs.

The major interactions listed by Drugs.com for amoxicillin and cephalexin were cross-verified using the DDInter application (Figure 4, chord diagrams). Notably, four out of the seven major interactions (57.1%) identified on Drugs.com were classified differently by DDInter: two were categorized as moderate, one interaction was not found (i.e., BCG vaccine), and one involved an item that was not included in the DDInter database (i.e., fecal microbiota spores, live). In the case of cephalexin, four out of six interactions (66.6%) identified on Drugs.com were classified differently by DDInter: two were categorized as moderate, one interaction was not found (i.e., BCG vaccine), and one involved an item that was not included in the DDInter database (i.e., fecal microbiota spores, live).

The heterogeneity of DDI online assessment tools, as demonstrated by significant differences in the displayed results, underscores the need for caution and awareness that these tools serve only as guidance mechanisms for therapeutic management. The most explicit recommendations are typically found in major interactions or contraindications. However, a notable disadvantage is that newly introduced antibiotics, such as contezolid, the cefepime-enmetazobactam combination, aztreonam-avibactam, and taurolidine, have not yet been included in most interaction checker databases. This omission is likely due to their novelty, which has not been matched by the speed of database updates, or because data on drug interactions for these agents are still limited. This situation underscores the imperative need for ongoing research in this area.

### Antibiotic-Antibiotic Combinations Targeting ESKAPE Pathogens

Antibiotic resistance constitutes a global threat due to its substantial economic impact and the significant burden it places on public health systems [147]. The ESKAPE nosocomial pathogens (i.e., *Enterococcus faecium*, *Staphylococcus aureus*, *Klebsiella pneumoniae*, *Acinetobacter baumannii*, *Pseudomonas aeruginosa*, and *Enterobacter* spp.) demonstrate multidrug resistance and virulence, representing a significant therapeutic challenge [148,149]. One of the still-effective strategies to counteract resistance mechanisms is the use of antibiotic combinations [137,150].

ACDB is a comprehensive online resource that compiles a vast array of antibiotic combinations, providing critical insights into their synergistic effects and potential applications in combating antibiotic resistance, in full agreement with the scientific literature [137].

To assess antibiotic combinations with potential for managing ESKAPE infections, searches were performed in ACDB based on the six bacterial species [151]. For each antibiotic combination, the fractional inhibitory concentration index (FICI) was calculated by dividing the MIC of each drug used in combination by its MIC when used alone. A lower FICI indicates a stronger interaction between the two drugs. Based on these values, interactions are categorized as synergistic, additive, antagonistic, or indifference-based [137,152].

The data presented reveal critical insights into the interactions of various antibiotic combinations against *Enterococcus faecium*. Notably, combinations involving fosfomycin with chloramphenicol and daptomycin demonstrate synergistic effects, with FICI values of 0.28 and 0.5, respectively. These values indicate that the combined use of these antibiotics enhances their antimicrobial efficacy beyond what is achievable with each drug alone, providing a potent strategy for combating infections caused by *Enterococcus faecium* [153]. However, the combination of vancomycin and D-cycloserine shows antagonism, as indicated by FICI values of 3 and 5 for distinct strains. This suggests that both medications hinder each other’s efficiency, potentially lowering the overall efficacy of the treatment. This antagonistic interaction underscores the significance of comprehending medication interactions to prevent poor combinations and enhance therapeutic techniques [154].

The analysis of antibiotic combinations revealed several notable synergistic interactions against methicillin-resistant *Staphylococcus aureus* (MRSA) and methicillin-sensitive *Staphylococcus aureus*. Fosfomycin demonstrated consistent synergy with other antibiotics, showing FICI values below 0.75 across various strains of MRSA. Notably, combinations with linezolid, gentamicin, and daptomycin exhibited strong synergistic effects with FICI values of 0.5 or lower. Similarly, fosfomycin combined with cefazolin displayed significant synergy in both methicillin-resistant and methicillin-sensitive strains, with FICI values ranging from 0.04 to 0.5 [155].

In methicillin-sensitive strains, fosfomycin consistently synergized with cefazolin, exhibiting FICI values between 0.25 and 0.5. These results underscore the potential of fosfomycin-based combinations in enhancing antibiotic efficacy against *Staphylococcal* infections [156].

Several antibiotic combinations showed significant synergistic effects against *Klebsiella pneumoniae*. Remarkably, fosfomycin combined with tigecycline demonstrated synergy with an FICI value of 0.25 [157]. Azithromycin paired with minocycline and cefixime also exhibited synergy, with FICI values of 0.38 [158]. Additionally, Doripenem combined with cefoxitin and tetracycline showed strong synergy, with FICI values of 0.38 and 0.14, respectively. Meropenem in combination with cefmetazole revealed synergy with an FICI value of 0.38 [159].

Colistin combined with meropenem exhibited strong synergy against *Acinetobacter baumannii* with an FICI value of 0.37 [160]. Chlorhexidine paired with meropenem, levofloxacin, or ciprofloxacin also demonstrated synergy, with FICI values of 0.25, 0.38, and 0.5, respectively [161]. Vancomycin combined with colistin showed consistent synergy with FICI values ranging from 0.16 to 0.28 [162]. These combinations may enhance therapeutic options against *Acinetobacter baumannii*.

The data reveal several important synergistic interactions among antibiotic combinations against *Pseudomonas aeruginosa*. Noteworthy synergies include cefepime with tobramycin, with an FICI of 0.5 [163] and combinations identified through Bliss indices: amikacin with cefotaxime (−0.33), cefsulodin with aztreonam (−0.55), ciprofloxacin with moxifloxacin (−0.24), fosfomycin with nitrofurantoin (−0.22), polymyxin b with erythromycin (−0.1), and polymyxin b with doxorubicin (−0.13). Additional synergistic pairs are polymyxin b with chlorhexidine (−0.62), minocycline with colistin (−0.34), chlorhexidine with colistin (−0.83), and chlorhexidine with moxifloxacin (−0.63) [164].

Synergistic interactions were observed between several antibiotic combinations against *Enterobacter cloacae* and its multidrug-resistant strains. Relevant synergistic pairs include aztreonam with cefotaxime, with an FICI of 0.04 [165] and meropenem with cefmetazole, with an FICI of 0.2 for *Enterobacter hormaechei* [166]. Additionally, ampicillin/sulbactam combined with amikacin showed synergy, with an FICI of 0.5, in multidrug-resistant *Enterobacter cloacae* [167].

The observed synergies suggest possible treatment strategies for resistant infections. However, further research is needed to confirm clinical relevance and optimize therapeutic use. Healthcare practitioners may improve patient safety and treatment efficacy by utilizing DDI-indicative online databases to identify and prevent potentially hazardous interactions. This e-health approach not only decreases the potential dangers associated with antibiotic use but also supports the overall goals of antibiotic management, ultimately resulting in improved medical results for patients receiving antibiotic treatment.

## 6. Future Perspectives in Antibiotic-Drug Interactions

Some of the future research directions aimed at improving the current management of antibiotic use focus on the discovery of new active compounds (e.g., bacteriophage proteins-guided therapy [168], peptide-based antibacterial compounds [169], nanomedical approaches through antibacterial nanoparticles [170], CRISPR-Cas systems using conjugative plasmids [171], etc.) or on the exploration of new possible combinations with antibacterial effects [172,173].

The development and design of these compounds inherently involve the risk of potential interactions, making drug-target interaction prediction essential for drug development and drug repurposing, particularly those methods based on machine learning [174]. Recently, advanced DDI prediction models, leveraging deep learning, have been categorized into four distinct groups: graph neural networks, multimodal strategies, knowledge graph frameworks, and neural network approaches [175]. Enhancements in the precision of these models are anticipated to lead to a decrease in the occurrence of the actual antibiotic-drug interactions.

The exploration of these sophisticated prediction tools and their applications must be supported by continuous research, particularly through clinical trials. Numerous clinical trials across various phases, types, and statuses highlight the ongoing need to recognize the importance and clinical impact of drug interactions, whether through the development of new active molecules, drug repurposing, or studies focused on safety in complex scenarios. This relevance is further evidenced by the studies listed in the ClinicalTrials.gov database [176]. A search for ‘antibiotic-drug interaction’ in the ‘other terms’ section, without applying additional filters, identified 570 studies (i.e., 29 terminated, 378 completed, 17 actives, not recruiting), with 518 being interventional (i.e., 3 in early phase 1, 269 in phase 1, 75 in phase 2, 54 in phase 3, 50 in phase 4, and 67 not applicable). To refine the results and focus on the most recent data, filters were applied for studies conducted from 1 January 2020 to 8 January 2024, and phase 4 interventional studies were selected, resulting in eight studies. Table 4 presents detailed information for seven of these eight studies, as one study (i.e., NCT04828824) was excluded due to its lack of relevance to the subject under evaluation.

## 7. Conclusions

The present narrative review provides an updated and comprehensive synthesis of the current state of knowledge regarding antibiotic-drug interactions, which are less studied than other DDIs due to short-term use of antibacterial agents. It offers an overview of antibiotics used in current medical practice, highlights clinically relevant interactions where antibiotics may act as perpetrator drugs, and discusses strategies to mitigate these risks. The geriatric population has specific characteristics that may increase the risk of interactions, being a group with heightened susceptibility to infections and consequently a greater need for antibiotic therapy. Furthermore, the study of potential antibiotic-antibiotic interactions is also essential, particularly in the context where one therapeutic approach for resistant bacteria involves the combination of multiple antibiotics.

The complexity and variety of interactions arising from pharmacokinetic or pharmacodynamic mechanisms can be challenging. Therefore, technological advancements have led to the development of online tools to identify potential drug interactions. However, these tools are primarily for guidance and provide information that must be clinically validated, with patients requiring careful monitoring.

## Figures and Tables

**Table 1:** Antibiotic-drug interactions involving mechanistic insights and recommendations.

Perpetrator Drug (Class)	Victim Drug	Effect and Mechanisms	Clinical Implications/ Recommendations	Ref.
Ciprofloxacin (quinolones)	Theophylline	effects of theophylline are increased by ciprofloxacin through the inhibition of CYP2D6-mediated metabolism of theophylline	ciprofloxacin-induced theophylline toxicity/decrease dosage and closely observe for signs of theophylline toxicity	[59,63]
	Erythromycin	additive effect with a prolongation of the QT interval	risk for torsades de pointes/avoid combination	[59,64]
	Voriconazole			
	Formoterol			
	Methadone	effects of methadone are increased by ciprofloxacin through the inhibition of CYP1A2-, CYP3A4-, CYP2D6-mediated metabolism of methadone	possible increased opioid effects/avoid opioid premedication when antibiotics are used for prophylaxis	[65]
Levofloxacin (quinolones)	Ciclosporin	increased concentrations of ciclosporin through the inhibition of CYP3A4-mediated metabolism of ciclosporin	risk for renal toxicity and neurotoxicity/monitor the function of the kidneys and the plasma concentrations	[66]
	Insulin	modified insulin needs	hypoglycemia or hyperglycemia/frequent monitoring of blood sugar levels	[67]
	Warfarin	increased concentrations of warfarin through the inhibition of CYP2C9-mediated metabolism of warfarin	possible increases of the INR values/INR monitoring	[68]
Neomycin (aminoglycosides)	Methotrexate	decreases the gastrointestinal absorption of oral methotrexate by half	lack of effect/2-4 h interval between administration	[64]
	Digoxin	decreases the gastrointestinal absorption of digoxin	lack of effect/2-4 h interval between administration	[69]
Meropenem Ertapenem Imipenem Doripenem (carbapenems, β-lactam)	Valproic acid	decreases in the concentration of valproic acid through the inhibition of valproate glucuronide hydrolysis, induction of valproate hepatic glucuronidation, and increases of the renal clearance of valproate glucuronide	lack of effect/valproic acid levels monitoring	[70,71]
Clarithromycin (macrolides)	Colchicine	increased concentrations of colchicine through the inhibition of P-glycoprotein and CYP3A4-mediated metabolism of colchicine	colchicine toxicity through gastrointestinal symptoms, multiple organ failure, and blood dyscrasias/use of another antibiotic	[72]
	Venlafaxine	additive effect with a prolongation of the QT interval	risk for torsades de pointes/avoid combination	[64]
	Terfenadine			
	Propafenone			
Telithromycin (macrolides)	Sotalol	additive effect with a prolongation of the QT interval	risk for torsades de pointes/avoid combination	[73]
	Voriconazole	increased concentrations of voriconazole through the inhibition of CYP3A4-mediated metabolism of voriconazole	risk for toxic effects/monitor liver functionality	[64]
	Midazolam	increased concentrations of midazolam through the inhibition of CYP3A4-mediated metabolism of midazolam	risk for toxic effects/reduce the dose of midazolam by half	[74]
Piperacillin (penicillins, β-lactam)	Depolarizing and non-depolarizing muscle relaxants	enhanced the effect of muscle relaxants through a neuromuscular blocking activity	risk for toxic effects/monitor the neuromuscular blockade	[64]
Rifampicin (rifamycins)	Amiodarone	decreased concentrations of amiodarone through the induction of CYP3A4-mediated metabolism of amiodarone	ineffective reaction to amiodarone/monitor the possible poor response, dose adjustments	[75]
	Paracetamol	decreased paracetamol concentration through the increase of paracetamol glucuronidation	lack of effect/use an alternative analgesic	[64]
	Cabazitaxel	competitive antagonism for UGT	lack of effect/avoid combination	[76,77]
Co-trimoxazole * (sulphonamides)	Sacubitril/Valsartan	additive effect on renal potassium regulation reducing aldosterone levels and potassium excretion	hyperkalemia/avoid combination	[78]
	Warfarin	increased concentrations of warfarin through the inhibition of CYP-mediated metabolism of warfarin	increased risk of bleeding/avoid combination	[79]
Vancomycin (glycopeptide)	Depolarizing and non-depolarizing muscle relaxants	enhanced the effect of muscle relaxants through a neuromuscular blocking activity	risk for toxic effects/monitor the neuromuscular blockade	[64]
Tetracycline (tetracyclines)	Methotrexate	increased concentration of methotrexate due to the disruption of the bacterial colonies involved in the metabolism of methotrexate	risk for toxic effects exerted by methotrexate/avoid combination	[80]
Chloramphenicol	Sulfonylureas as antidiabetic agents	increased concentrations of sulphonylureas through the inhibition of CYP2C9-mediated metabolism of sulphonylureas	elevated risk of hypoglycemia/monitor the blood sugar level	[81]
	Vitamin B12	decrease in the efficacy of vitamin B12 due to the inhibition of the bone marrow	risks related to the inhibition of bone marrow function/monitor the levels of vitamin B12 and the full blood count	[64]
Quinupristin/dalfopristin (streptogramins)	Atomoxetine	additive effect with a prolongation of the QT interval	risk for torsades de pointes/avoid combination	[64]
	Sotalol			
	Disopyramide			
Amoxicillin (aminopenicillin, β-lactam)	Warfarin	probably through the reduction of intestinal bacteria that produce vitamin K, leading to a shortage of vitamin K	possible increases of the INR values/INR monitoring	[73]
	Acenocumarol			
Linezolid	Fentanyl	uncertain, possibly due to the competitive inhibition of monoamine oxidase-A	risk for serotonin syndrome/avoid combination	[82]

*, trimethoprim-sulfamethoxazole; INR, International Normalized Ratio; UGT, uridine 5′-diphospho-glucuronosyltransferase.

**Table 2:** Major antibiotic-drug interactions identified via Drugs.com.

Antibiotic	Drugs and Vaccines with Major Interaction Potential in Combination with the Antibiotic
Ciprofloxacin	Acalabrutinib, acetohexamide, adagrasib, aminolevulinic acid, aminophylline, amiodarone, amisulpride, anagrelide, anisindione, arsenic trioxide, avanafil, avapritinib, BCG vaccine, bedaquiline, bempedoic acid, bepridil, betamethasone, bosutinib, brexpiprazole, brigatinib, bromocriptine, bupropion, butorphanol, cabozantinib, capivasertib, ceritinib, chloroquine, chlorpropamide, cholera vaccine (live), cisapride, citalopram, clozapine, cobimetinib, colchicine, cortisone, crizotinib, deflazacort, dexamethasone, dicumarol, disopyramide, dofetilide, dolasetron, dronedarone, droperidol, duloxetine, efavirenz, elacestrant, eliglustat, entrectinib, eplerenone, escitalopram, etrasimod, fecal microbiota spores (live), fenfluramine, fexinidazole, fezolinetant, finerenone, fingolimod, flibanserin, fludrocortisone, gepirone, glimepiride, glipizide, glyburide, guanfacine, halofantrine, haloperidol, hydrocodone, hydrocortisone, hydroxychloroquine, ibrutinib, ibutilide, iloperidone, infigratinib, insulin, insulin aspart, insulin aspart protamine, insulin degludec, insulin detemir, insulin glargine, insulin glulisine, insulin inhalation (rapid-acting), insulin isophane, insulin lispro, insulin lispro protamine, insulin regular, insulin zinc, insulin zinc extended, iohexol, iomeprol, iopamidol, ivabradine, ivosidenib, lefamulin, lemborexant, levoketoconazole, levomethadylacetate, lomitapide, lonafarnib, lumateperone, lurbinectedin, mavacamten, mavorixafor, mesoridazine, methadone, methylprednisolone, metrizamide, mifepristone, mobocertinib, mycophenolate mofetil, mycophenolic acid, naloxegol, nateglinide, neratinib, nilotinib, nirogacestat, olanzapine, olaparib, oliceridine, oxtriphylline, oxycodone, ozanimod, pacritinib, palovarotene, panobinostat, papaverine, pasireotide, pemigatinib, pexidartinib, pimozide, pirfenidone, ponesimod, prednisolone, prednisone, procainamide, quinidine, quizartinib, rasagiline, repaglinide, repotrectinib, ribociclib, saquinavir, selpercatinib, selumetinib, siponimod, sirolimus protein-bound, sonidegib, sotalol, suvorexant, tasimelteon, tazemetostat, theophylline, thioridazine, tizanidine, tolazamide, tolbutamide, toremifene, tramadol, triamcinolone, typhoid vaccine (live), vamorolone, vandetanib, vemurafenib, venetoclax, voclosporin, warfarin, zanubrutinib, ziprasidone, zolpidem
Trimethoprim	Amiloride, azilsartan medoxomil, BCG vaccine, benazepril, candesartan, captopril, cholera vaccine (live), dofetilide, enalapril, enalaprilat, eplerenone, eprosartan, fecal microbiota spores (live), finerenone, fosinopril, fosphenytoin, irbesartan, leucovorin, levoleucovorin, lisinopril, losartan, lvp solution with potassium, methotrexate, moexipril, olmesartan, parenteral nutrition solution w/electrolytes, perindopril, phenytoin, potassium acetate, potassium acid phosphate, potassium bicarbonate, potassium chloride, potassium citrate, potassium gluconate, potassium iodide, potassium perchlorate, potassium phosphate, prilocaine, procaine penicillin, quinapril, ramipril, spironolactone, telmisartan, trandolapril, triamterene, typhoid vaccine (live), valsartan
Amoxicillin	BCG vaccine, cholera vaccine (live), fecal microbiota spores (live), methotrexate, mycophenolate mofetil, mycophenolic acid, typhoid vaccine (live)
Cephalexin	BCG vaccine, cholera vaccine (live), fecal microbiota spores (live), mycophenolate mofetil, mycophenolic acid, typhoid vaccine (live)
Azithromycin	Adagrasib, amiodarone, amisulpride, anagrelide, arsenic trioxide, BCG vaccine, bedaquiline, bepridil, berotralstat, betrixaban, cabozantinib, ceritinib, chloroquine, cholera vaccine (live), cisapride, citalopram, clozapine, colchicine, crizotinib, disopyramide, dofetilide, dolasetron, dronedarone, droperidol, edoxaban, efavirenz, escitalopram, etrasimod, fecal microbiota spores (live), fexinidazole, fingolimod, gatifloxacin, grepafloxacin, halofantrine, haloperidol, hydroxychloroquine, ibutilide, iloperidone, ivabradine, ivosidenib, lefamulin, leflunomide, levoketoconazole, levomethadyl acetate, lomitapide, mavorixafor, mesoridazine, methadone, mifepristone, mipomersen, mobocertinib, morphine, moxifloxacin, mycophenolate mofetil, mycophenolic acid, nilotinib, osimertinib, ozanimod, pacritinib, panobinostat, papaverine, pasireotide, pazopanib, pexidartinib, pimozide, ponesimod, procainamide, quinidine, quizartinib, relugolix, ribociclib, saquinavir, selpercatinib, siponimod, sotalol, sparfloxacin, teriflunomide, thioridazine, toremifene, typhoid vaccine (live), vandetanib, vemurafenib, ziprasidone

BCG, bacillus of Calmette and Guérin.

**Table 3:** Interactions with different status generated by Medscape, WebMD, and DrugBank versus Drugs.com.

Antibiotic	Drug Pair	Results Displayed
Ciprofloxacin *	Acetohexamide, anisindione, bepridil, dicumarol, halofantrine, infigratinib, insulin, insulin lispro protamine, insulin zinc, insulin zinc extended, iohexol, iomeprol, iopamidol, levoketoconazole, levomethadyl acetate, mesoridazine, mycophenolate mofetil, mycophenolic acid, oxtriphylline, panobinostat	No results
	Acalabrutinib, adagrasib, avanafil, bempedoic acid, betamethasone, brexpiprazole, brigatinib, bromocriptine, bupropion, butorphanol, cabozantinib, capivasertib, cisapride, deflazacort, elacestrant, eplerenone, fenfluramine, guanfacine, hydrocodone, hydrocortisone, insulin inhalation, insulin aspart protamine, insulin degludec, insulin detemir, insulin glargine, insulin glulisine, insulin isophane, ivabradine, lefamulin, lumateperone, lurbinectedin, mavacamten, metrizamide, naloxegol, neratinib, nirogacestat, oliceridine, oxycodone, pacritinib, palovarotene, papaverine, pemigatinib, pexidartinib, ponesimod, repotrectinib, selumetinib, sirolimus protein-bound, sonidegib, suvorexant, tasimelteon, tazemetostat, tramadol, triamcinolone, vamorolone, zanubrutinib	No Interactions Found
	Amiodarone, arsenic trioxide, avapritinib, bedaquiline, bosutinib, chloroquine, chlorpropamide, citalopram, colchicine, cortisone, crizotinib, dexamethasone, disopyramide, dolasetron, droperidol, duloxetine, efavirenz, escitalopram, etrasimod, finerenone, fingolimod, fludrocortisone, gepirone, glimepiride, glipizide, glyburide, haloperidol, ibutilide, iloperidone, insulin aspart, insulin lispro, insulin regular, lemborexant, lomitapide, mavorixafor, methadone, methylprednisolone, mifepristone, nateglinide, nilotinib, ozanimod, pasireotide, pimozide, prednisolone, prednisone, procainamide, quinidine, quizartinib, repaglinide, selpercatinib, sotalol, thioridazine, tolazamide, tolbutamide, voclosporin, warfarin, ziprasidone, zolpidem	Monitor closely (moderate)
Azithromycin **	Bepridil, grepafloxacin, halofantrine, levomethadyl acetate, mesoridazine, mycophenolate mofetil, mycophenolic acid, sparfloxacin	No results
	Cabozantinib, etrasimod, gatifloxacin, ivabradine, ivosidenib, leflunomide, lomitapide, mipomersen, morphine, pacritinib, papaverine, pexidartinib, ponesimod, relugolix, teriflunomide	No Interactions Found
	Amiodarone, bedaquiline, berotralstat, betrixaban, chloroquine, citalopram, crizotinib, disopyramide, droperidol, edoxaban, haloperidol, ibutilide, levoketoconazole, mavorixafor, mifepristone, moxifloxacin, nilotinib, osimertinib, ozanimod, pasireotide, procainamide, quinidine, quizartinib, selpercatinib, sotalol, thioridazine, ziprasidone	Monitor closely (moderate)
	Dolasetron, iloperidone, methadone, pazopanib	Minor
Trimethoprim ***	LVP solution with potassium, parenteral nutrition solution w/electrolytes, potassium perchlorate	No results
	BCG vaccine, cholera vaccine, fecal microbiota spores, leucovorin, levoleucovorin, prilocaine, typhoid vaccine	No Interactions Found
	Azilsartan medoxomil, candesartan, eprosartan, finerenone, fosinopril, fosphenytoin, irbesartan, losartan, olmesartan, phenytoin, potassium acetate, potassium acid phosphate, potassium bicarbonate, potassium chloride, potassium citrate, potassium gluconate, potassium iodide, potassium phosphate, spironolactone, telmisartan, valsartan	Monitor closely (moderate)
	Amiloride, benazepril, captopril, enalapril, enalaprilat, eplerenone, lisinopril, moexipril, perindopril, procaine penicillin, quinapril, ramipril, trandolapril, triamterene	Minor

* Results obtained from the drug interaction checker tool from Medscape; ** results obtained from the drug interaction checker tool from WebMD; *** results obtained from the drug interaction checker tool from DrugBank.

**Table 4:** Overview of ongoing and completed clinical trials evaluating antibiotic-drug interactions from various perspectives.

Database ID	Official Title	Description	Condition	Recruitment Status
NCT04840862	Impact of Rifabutin on the Pharmacokinetics of Elexacaftor/Tezacaftor/Ivacaftor	A single-center, prospective, nonrandomized, open-label study was conducted in healthy adults to assess how Trikafta’s pharmacokinetics are affected by rifabutin	Drug-Drug Interaction	Completed
NCT04671589	Antidote for Valproic Acid Toxicity: a New Indication for Meropenem Antibiotic. A Randomized Placebo-controlled Trial	Evaluation of an antidote for VPA by leveraging the well-documented drug-drug interaction between VPA and carbapenems, which significantly reduces VPA serum levels during concurrent administration	Drug toxicity	Unknown
NCT04140903	Partial Oral Antibiotic Treatment for Bacterial Brain Abscess: An Open-label Randomized Non-inferiority Trial	Assessment of the clinical viability of oral antibiotics for the treatment of brain abscess	Brain abscess	Recruiting
NCT04551573	A Study of the Pharmacokinetic and Pharmacodynamic Interactions Between Bictegravir, Tenofovir Alafenamide and Rifapentine in Healthy Adult Subjects	A single-center, open-label investigation that examines the pharmacokinetic interaction between bictegravir and tenofovir alafenamide when administered with rifapentine	Tuberculosis	Withdrawn
NCT05588492	The Safety, Completion Rate and Prevention Effect by Rifamycin-containing Regimens for Latent Tuberculosis Infection in Patients With Kidney Transplantation: a Prospective Intervention Pilot Study	Evaluation of the co-administration of rifamycin and immunosuppressants in kidney transplant recipients	Pulmonology	Recruiting
NCT05046132	A Randomized, Double-Blind, 3-arm, Parallel Group, Placebo- and Positive-controlled Study to Investigate the Effects of Setmelanotide on QTc Interval in Healthy Subjects	An assessment of the effects of various concentrations of setmelanotide on the QT interval in a study that employed a double-blind, randomized design with positive and placebo controls	Healthy	Completed with results
NCT06178627	A Multi-center, Prospective, Randomized Trial of Amphotericin B in the Initial Antifungal Therapy for Non-HIV Cryptococcal Meningitis Patients	Comparative assessments to evaluate the safety and effectiveness of standard-dose amphotericin B (0.7 mg/kg/day) versus a lower dose (0.5 mg/kg/day) in the initial antifungal therapy for non-HIV patients with cryptococcal meningitis, since Asian patients may exhibit variations in drug metabolism and pharmacokinetics	Contraception Behavior	Enrolling by invitation

VPA, valproic acid.

## Data Availability

All the information provided in the manuscript is contained within the paper and is supported by the inserted references.
